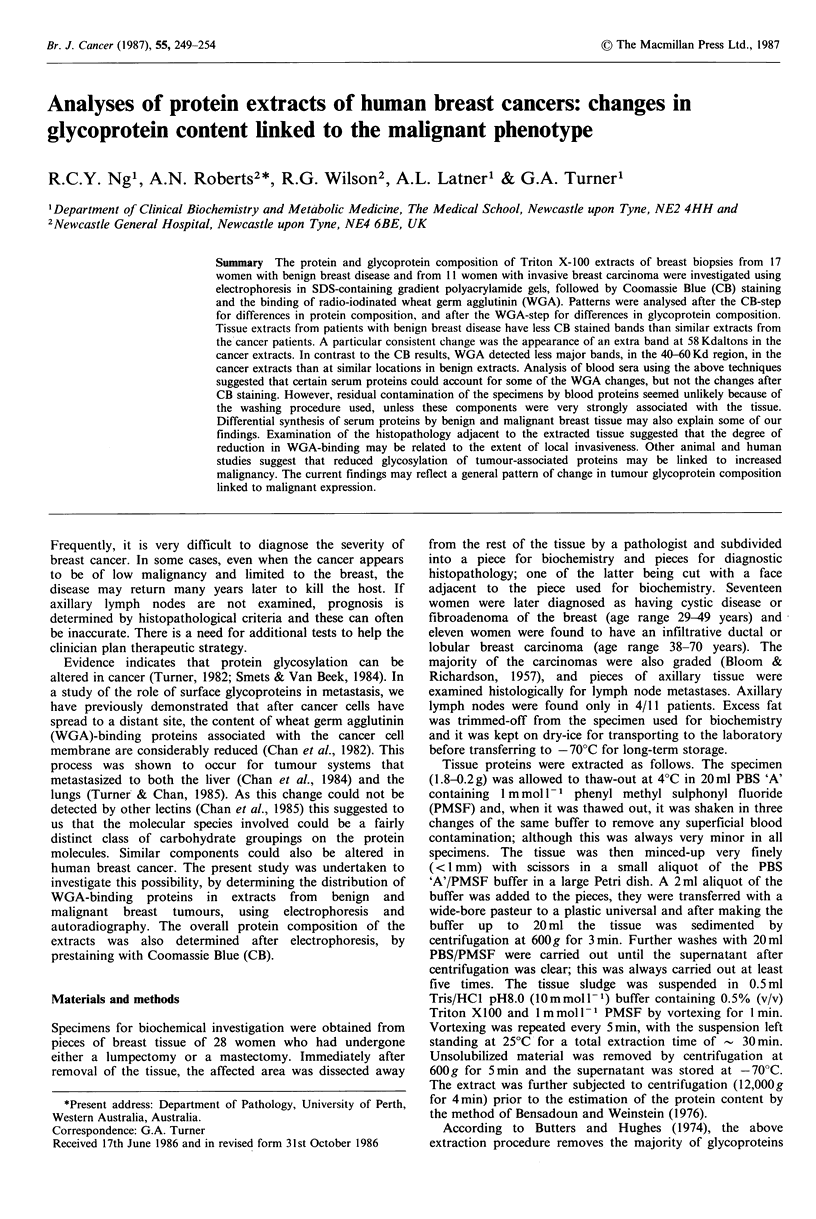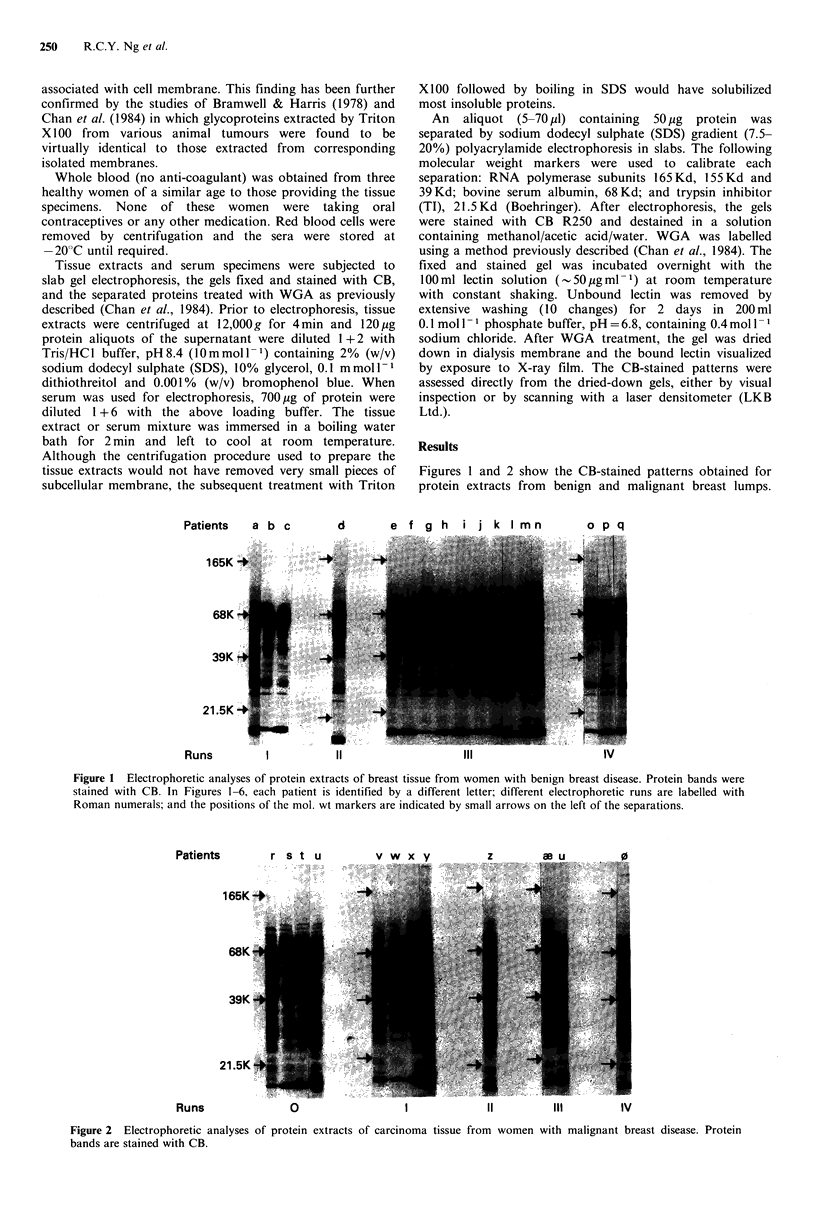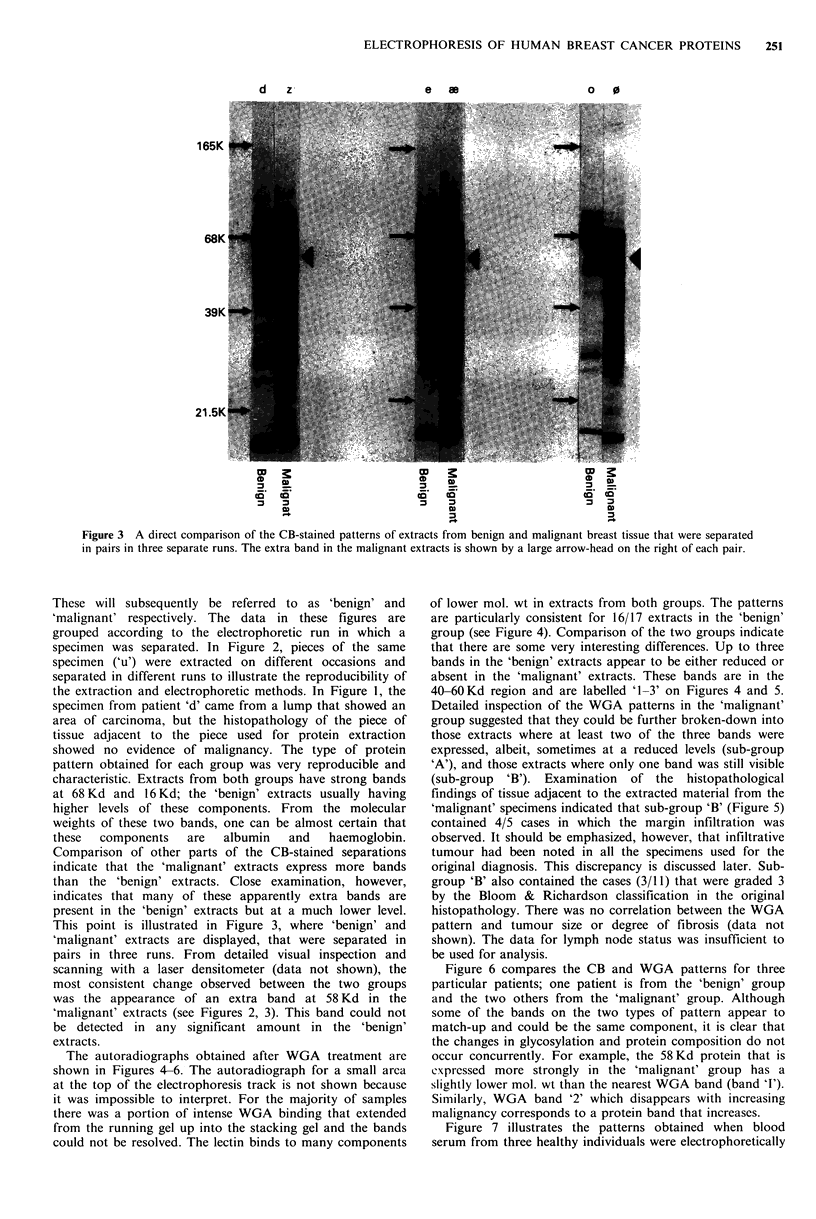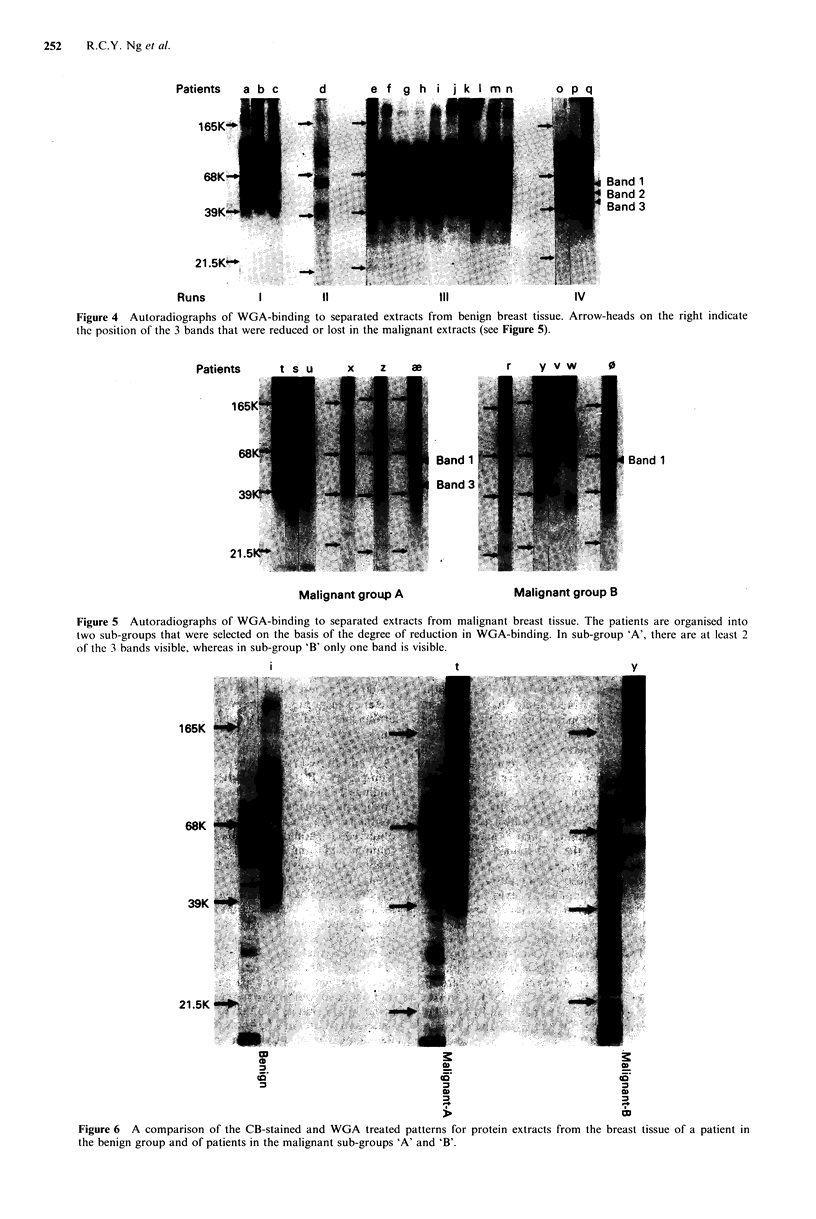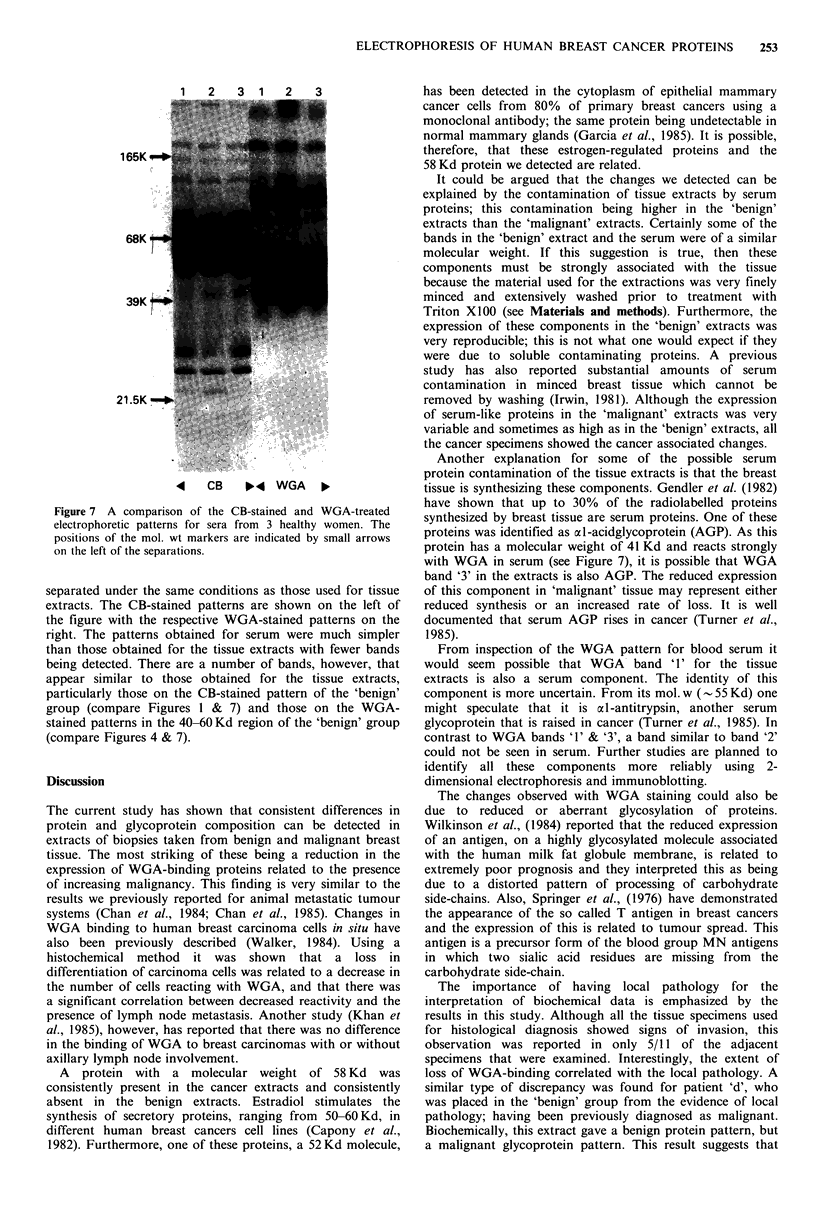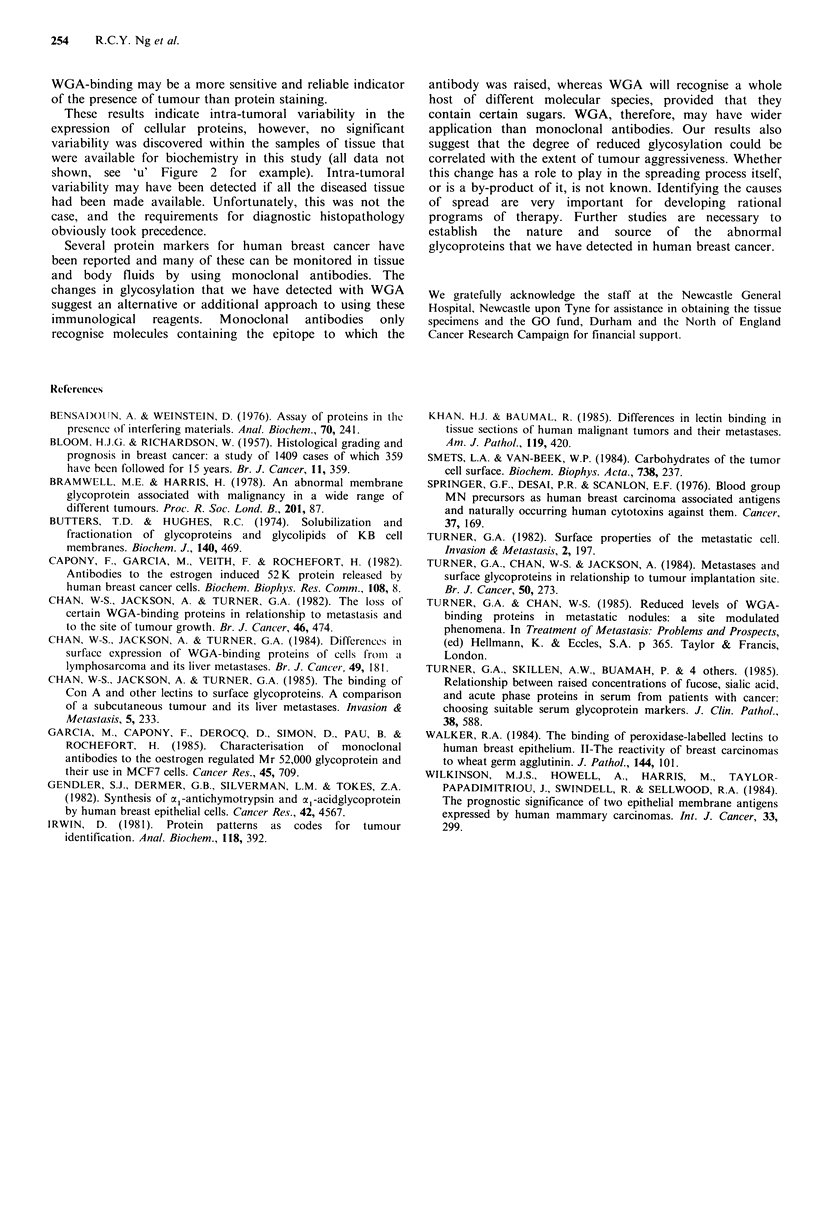# Analyses of protein extracts of human breast cancers: changes in glycoprotein content linked to the malignant phenotype.

**DOI:** 10.1038/bjc.1987.48

**Published:** 1987-03

**Authors:** R. C. Ng, A. N. Roberts, R. G. Wilson, A. L. Latner, G. A. Turner

## Abstract

**Images:**


*Br. J. Cancer* (1987), **55**, 249–254 © The Macmillan Press Ltd., 1987

### Analyses of protein extracts of human breast cancers: changes in glycoprotein content linked to the malignant phenotype

R.C.Y. Ng[1], A.N. Roberts[2]*, R.G. Wilson[2], A.L. Latner[1] & G.A. Turner[1]

[1]*Department of Clinical Biochemistry and Metabolic Medicine, The Medical School, Newcastle upon Tyne, NE2 4HH and* [2]*Newcastle General Hospital, Newcastle upon Tyne, NE4 6BE, UK*

**Summary** The protein and glycoprotein composition of Triton X-100 extracts of breast biopsies from 17 women with benign breast disease and from 11 women with invasive breast carcinoma were investigated using electrophoresis in SDS-containing gradient polyacrylamide gels, followed by Coomassie Blue (CB) staining and the binding of radio-iodinated wheat germ agglutinin (WGA). Patterns were analysed after the CB-step for differences in protein composition, and after the WGA-step for differences in glycoprotein composition. Tissue extracts from patients with benign breast disease have less CB stained bands than similar extracts from the cancer patients. A particular consistent change was the appearance of an extra band at 58 Kdaltons in the cancer extracts. In contrast to the CB results, WGA detected less major bands, in the 40–60 Kd region, in the cancer extracts than at similar locations in benign extracts. Analysis of blood sera using the above techniques suggested that certain serum proteins could account for some of the WGA changes, but not the changes after CB staining. However, residual contamination of the specimens by blood proteins seemed unlikely because of the washing procedure used, unless these components were very strongly associated with the tissue. Differential synthesis of serum proteins by benign and malignant breast tissue may also explain some of our findings. Examination of the histopathology adjacent to the extracted tissue suggested that the degree of reduction in WGA-binding may be related to the extent of local invasiveness. Other animal and human studies suggest that reduced glycosylation of tumour-associated proteins may be linked to increased malignancy. The current findings may reflect a general pattern of change in tumour glycoprotein composition linked to malignant expression.

Frequently, it is very difficult to diagnose the severity of breast cancer. In some cases, even when the cancer appears to be of low malignancy and limited to the breast, the disease may return many years later to kill the host. If axillary lymph nodes are not examined, prognosis is determined by histopathological criteria and these can often be inaccurate. There is a need for additional tests to help the clinician plan therapeutic strategy.

Evidence indicates that protein glycosylation can be altered in cancer (Turner, 1982; Smets & Van Beek, 1984). In a study of the role of surface glycoproteins in metastasis, we have previously demonstrated that after cancer cells have spread to a distant site, the content of wheat germ agglutinin (WGA)-binding proteins associated with the cancer cell membrane are considerably reduced (Chan *et al.*, 1982). This process was shown to occur for tumour systems that metastasized to both the liver (Chan *et al.*, 1984) and the lungs (Turner & Chan, 1985). As this change could not be detected by other lectins (Chan *et al.*, 1985) this suggested to us that the molecular species involved could be a fairly distinct class of carbohydrate groupings on the protein molecules. Similar components could also be altered in human breast cancer. The present study was undertaken to investigate this possibility, by determining the distribution of WGA-binding proteins in extracts from benign and malignant breast tumours, using electrophoresis and autoradiography. The overall protein composition of the extracts was also determined after electrophoresis, by prestaining with Coomassie Blue (CB).

### Materials and methods

Specimens for biochemical investigation were obtained from pieces of breast tissue of 28 women who had undergone either a lumpectomy or a mastectomy. Immediately after removal of the tissue, the affected area was dissected away from the rest of the tissue by a pathologist and subdivided into a piece for biochemistry and pieces for diagnostic histopathology; one of the latter being cut with a face adjacent to the piece used for biochemistry. Seventeen women were later diagnosed as having cystic disease or fibroadenoma of the breast (age range 29–49 years) and eleven women were found to have an infiltrative ductal or lobular breast carcinoma (age range 38–70 years). The majority of the carcinomas were also graded (Bloom & Richardson, 1957), and pieces of axillary tissue were examined histologically for lymph node metastases. Axillary lymph nodes were found only in 4/11 patients. Excess fat was trimmed-off from the specimen used for biochemistry and it was kept on dry-ice for transporting to the laboratory before transferring to −70°C for long-term storage.

Tissue proteins were extracted as follows. The specimen (1.8–0.2 g) was allowed to thaw-out at 4°C in 20 ml PBS 'A' containing $1\,mmol\,l^{-1}$ phenyl methyl sulphonyl fluoride (PMSF) and, when it was thawed out, it was shaken in three changes of the same buffer to remove any superficial blood contamination; although this was always very minor in all specimens. The tissue was then minced-up very finely (<1 mm) with scissors in a small aliquot of the PBS 'A'/PMSF buffer in a large Petri dish. A 2 ml aliquot of the buffer was added to the pieces, they were transferred with a wide-bore pasteur to a plastic universal and after making the buffer up to 20 ml the tissue was sedimented by centrifugation at 600 *g* for 3 min. Further washes with 20 ml PBS/PMSF were carried out until the supernatant after centrifugation was clear; this was always carried out at least five times. The tissue sludge was suspended in 0.5 ml Tris/HCl pH8.0 ($10\,mmol\,l^{-1}$) buffer containing 0.5% (v/v) Triton X100 and $1\,mmol\,l^{-1}$ PMSF by vortexing for 1 min. Vortexing was repeated every 5 min, with the suspension left standing at 25°C for a total extraction time of ~ 30 min. Unsolubilized material was removed by centrifugation at 600 *g* for 5 min and the supernatant was stored at −70°C. The extract was further subjected to centrifugation (12,000 *g* for 4 min) prior to the estimation of the protein content by the method of Bensadoun and Weinstein (1976).

According to Butters and Hughes (1974), the above extraction procedure removes the majority of glycoproteins

*Present address: Department of Pathology, University of Perth, Western Australia, Australia.

Correspondence: G.A. Turner

Received 17th June 1986 and in revised form 31st October 1986

associated with cell membrane. This finding has been further confirmed by the studies of Bramwell & Harris (1978) and Chan *et al.* (1984) in which glycoproteins extracted by Triton X100 from various animal tumours were found to be virtually identical to those extracted from corresponding isolated membranes.

Whole blood (no anti-coagulant) was obtained from three healthy women of a similar age to those providing the tissue specimens. None of these women were taking oral contraceptives or any other medication. Red blood cells were removed by centrifugation and the sera were stored at −20°C until required.

Tissue extracts and serum specimens were subjected to slab gel electrophoresis, the gels fixed and stained with CB, and the separated proteins treated with WGA as previously described (Chan *et al.*, 1984). Prior to electrophoresis, tissue extracts were centrifuged at 12,000*g* for 4 min and 120 μg protein aliquots of the supernatant were diluted 1+2 with Tris/HCl buffer, pH 8.4 (10 mmol $l^{-1}$) containing 2% (w/v) sodium dodecyl sulphate (SDS), 10% glycerol, 0.1 mmol $l^{-1}$ dithiothreitol and 0.001% (w/v) bromophenol blue. When serum was used for electrophoresis, 700 μg of protein were diluted 1+6 with the above loading buffer. The tissue extract or serum mixture was immersed in a boiling water bath for 2 min and left to cool at room temperature. Although the centrifugation procedure used to prepare the tissue extracts would not have removed very small pieces of subcellular membrane, the subsequent treatment with Triton X100 followed by boiling in SDS would have solubilized most insoluble proteins.

An aliquot (5–70 μl) containing 50 μg protein was separated by sodium dodecyl sulphate (SDS) gradient (7.5–20%) polyacrylamide electrophoresis in slabs. The following molecular weight markers were used to calibrate each separation: RNA polymerase subunits 165 Kd, 155 Kd and 39 Kd; bovine serum albumin, 68 Kd; and trypsin inhibitor (TI), 21.5 Kd (Boehringer). After electrophoresis, the gels were stained with CB R250 and destained in a solution containing methanol/acetic acid/water. WGA was labelled using a method previously described (Chan *et al.*, 1984). The fixed and stained gel was incubated overnight with the 100 ml lectin solution (~50 μg ml$^{-1}$) at room temperature with constant shaking. Unbound lectin was removed by extensive washing (10 changes) for 2 days in 200 ml 0.1 mol $l^{-1}$ phosphate buffer, pH = 6.8, containing 0.4 mol $l^{-1}$ sodium chloride. After WGA treatment, the gel was dried down in dialysis membrane and the bound lectin visualized by exposure to X-ray film. The CB-stained patterns were assessed directly from the dried-down gels, either by visual inspection or by scanning with a laser densitometer (LKB Ltd.).

### Results

Figures 1 and 2 show the CB-stained patterns obtained for protein extracts from benign and malignant breast lumps.

**Figure 1** Electrophoretic analyses of protein extracts of breast tissue from women with benign breast disease. Protein bands were stained with CB. In Figures 1–6, each patient is identified by a different letter; different electrophoretic runs are labelled with Roman numerals; and the positions of the mol. wt markers are indicated by small arrows on the left of the separations.

**Figure 2** Electrophoretic analyses of protein extracts of carcinoma tissue from women with malignant breast disease. Protein bands are stained with CB.

**Figure 3** A direct comparison of the CB-stained patterns of extracts from benign and malignant breast tissue that were separated in pairs in three separate runs. The extra band in the malignant extracts is shown by a large arrow-head on the right of each pair.

These will subsequently be referred to as 'benign' and 'malignant' respectively. The data in these figures are grouped according to the electrophoretic run in which a specimen was separated. In Figure 2, pieces of the same specimen ('u') were extracted on different occasions and separated in different runs to illustrate the reproducibility of the extraction and electrophoretic methods. In Figure 1, the specimen from patient 'd' came from a lump that showed an area of carcinoma, but the histopathology of the piece of tissue adjacent to the piece used for protein extraction showed no evidence of malignancy. The type of protein pattern obtained for each group was very reproducible and characteristic. Extracts from both groups have strong bands at 68 Kd and 16 Kd; the 'benign' extracts usually having higher levels of these components. From the molecular weights of these two bands, one can be almost certain that these components are albumin and haemoglobin. Comparison of other parts of the CB-stained separations indicate that the 'malignant' extracts express more bands than the 'benign' extracts. Close examination, however, indicates that many of these apparently extra bands are present in the 'benign' extracts but at a much lower level. This point is illustrated in Figure 3, where 'benign' and 'malignant' extracts are displayed, that were separated in pairs in three runs. From detailed visual inspection and scanning with a laser densitometer (data not shown), the most consistent change observed between the two groups was the appearance of an extra band at 58 Kd in the 'malignant' extracts (see Figures 2, 3). This band could not be detected in any significant amount in the 'benign' extracts.

The autoradiographs obtained after WGA treatment are shown in Figures 4–6. The autoradiograph for a small area at the top of the electrophoresis track is not shown because it was impossible to interpret. For the majority of samples there was a portion of intense WGA binding that extended from the running gel up into the stacking gel and the bands could not be resolved. The lectin binds to many components of lower mol. wt in extracts from both groups. The patterns are particularly consistent for 16/17 extracts in the 'benign' group (see Figure 4). Comparison of the two groups indicate that there are some very interesting differences. Up to three bands in the 'benign' extracts appear to be either reduced or absent in the 'malignant' extracts. These bands are in the 40–60 Kd region and are labelled '1–3' on Figures 4 and 5. Detailed inspection of the WGA patterns in the 'malignant' group suggested that they could be further broken-down into those extracts where at least two of the three bands were expressed, albeit, sometimes at a reduced levels (sub-group 'A'), and those extracts where only one band was still visible (sub-group 'B'). Examination of the histopathological findings of tissue adjacent to the extracted material from the 'malignant' specimens indicated that sub-group 'B' (Figure 5) contained 4/5 cases in which the margin infiltration was observed. It should be emphasized, however, that infiltrative tumour had been noted in all the specimens used for the original diagnosis. This discrepancy is discussed later. Sub-group 'B' also contained the cases (3/11) that were graded 3 by the Bloom & Richardson classification in the original histopathology. There was no correlation between the WGA pattern and tumour size or degree of fibrosis (data not shown). The data for lymph node status was insufficient to be used for analysis.

Figure 6 compares the CB and WGA patterns for three particular patients; one patient is from the 'benign' group and the two others from the 'malignant' group. Although some of the bands on the two types of pattern appear to match-up and could be the same component, it is clear that the changes in glycosylation and protein composition do not occur concurrently. For example, the 58 Kd protein that is expressed more strongly in the 'malignant' group has a slightly lower mol. wt than the nearest WGA band (band '1'). Similarly, WGA band '2' which disappears with increasing malignancy corresponds to a protein band that increases.

Figure 7 illustrates the patterns obtained when blood serum from three healthy individuals were electrophoretically

**Figure 4** Autoradiographs of WGA-binding to separated extracts from benign breast tissue. Arrow-heads on the right indicate the position of the 3 bands that were reduced or lost in the malignant extracts (see **Figure 5**).

**Figure 5** Autoradiographs of WGA-binding to separated extracts from malignant breast tissue. The patients are organised into two sub-groups that were selected on the basis of the degree of reduction in WGA-binding. In sub-group 'A', there are at least 2 of the 3 bands visible, whereas in sub-group 'B' only one band is visible.

**Figure 6** A comparison of the CB-stained and WGA treated patterns for protein extracts from the breast tissue of a patient in the benign group and of patients in the malignant sub-groups 'A' and 'B'.

**Figure 7** A comparison of the CB-stained and WGA-treated electrophoretic patterns for sera from 3 healthy women. The positions of the mol. wt markers are indicated by small arrows on the left of the separations.

separated under the same conditions as those used for tissue extracts. The CB-stained patterns are shown on the left of the figure with the respective WGA-stained patterns on the right. The patterns obtained for serum were much simpler than those obtained for the tissue extracts with fewer bands being detected. There are a number of bands, however, that appear similar to those obtained for the tissue extracts, particularly those on the CB-stained pattern of the 'benign' group (compare Figures 1 & 7) and those on the WGA-stained patterns in the 40–60 Kd region of the 'benign' group (compare Figures 4 & 7).

### Discussion

The current study has shown that consistent differences in protein and glycoprotein composition can be detected in extracts of biopsies taken from benign and malignant breast tissue. The most striking of these being a reduction in the expression of WGA-binding proteins related to the presence of increasing malignancy. This finding is very similar to the results we previously reported for animal metastatic tumour systems (Chan *et al*., 1984; Chan *et al*., 1985). Changes in WGA binding to human breast carcinoma cells *in situ* have also been previously described (Walker, 1984). Using a histochemical method it was shown that a loss in differentiation of carcinoma cells was related to a decrease in the number of cells reacting with WGA, and that there was a significant correlation between decreased reactivity and the presence of lymph node metastasis. Another study (Khan *et al*., 1985), however, has reported that there was no difference in the binding of WGA to breast carcinomas with or without axillary lymph node involvement.

A protein with a molecular weight of 58 Kd was consistently present in the cancer extracts and consistently absent in the benign extracts. Estradiol stimulates the synthesis of secretory proteins, ranging from 50–60 Kd, in different human breast cancers cell lines (Capony *et al*., 1982). Furthermore, one of these proteins, a 52 Kd molecule, has been detected in the cytoplasm of epithelial mammary cancer cells from 80% of primary breast cancers using a monoclonal antibody; the same protein being undetectable in normal mammary glands (Garcia *et al*., 1985). It is possible, therefore, that these estrogen-regulated proteins and the 58 Kd protein we detected are related.

It could be argued that the changes we detected can be explained by the contamination of tissue extracts by serum proteins; this contamination being higher in the 'benign' extracts than the 'malignant' extracts. Certainly some of the bands in the 'benign' extract and the serum were of a similar molecular weight. If this suggestion is true, then these components must be strongly associated with the tissue because the material used for the extractions was very finely minced and extensively washed prior to treatment with Triton X100 (see **Materials and methods**). Furthermore, the expression of these components in the 'benign' extracts was very reproducible; this is not what one would expect if they were due to soluble contaminating proteins. A previous study has also reported substantial amounts of serum contamination in minced breast tissue which cannot be removed by washing (Irwin, 1981). Although the expression of serum-like proteins in the 'malignant' extracts was very variable and sometimes as high as in the 'benign' extracts, all the cancer specimens showed the cancer associated changes.

Another explanation for some of the possible serum protein contamination of the tissue extracts is that the breast tissue is synthesizing these components. Gendler *et al*. (1982) have shown that up to 30% of the radiolabelled proteins synthesized by breast tissue are serum proteins. One of these proteins was identified as $\alpha$1-acidglycoprotein (AGP). As this protein has a molecular weight of 41 Kd and reacts strongly with WGA in serum (see Figure 7), it is possible that WGA band '3' in the extracts is also AGP. The reduced expression of this component in 'malignant' tissue may represent either reduced synthesis or an increased rate of loss. It is well documented that serum AGP rises in cancer (Turner *et al*., 1985).

From inspection of the WGA pattern for blood serum it would seem possible that WGA band '1' for the tissue extracts is also a serum component. The identity of this component is more uncertain. From its mol. w ($\sim$55 Kd) one might speculate that it is $\alpha$1-antitrypsin, another serum glycoprotein that is raised in cancer (Turner *et al*., 1985). In contrast to WGA bands '1' & '3', a band similar to band '2' could not be seen in serum. Further studies are planned to identify all these components more reliably using 2-dimensional electrophoresis and immunoblotting.

The changes observed with WGA staining could also be due to reduced or aberrant glycosylation of proteins. Wilkinson *et al*., (1984) reported that the reduced expression of an antigen, on a highly glycosylated molecule associated with the human milk fat globule membrane, is related to extremely poor prognosis and they interpreted this as being due to a distorted pattern of processing of carbohydrate side-chains. Also, Springer *et al*., (1976) have demonstrated the appearance of the so called T antigen in breast cancers and the expression of this is related to tumour spread. This antigen is a precursor form of the blood group MN antigens in which two sialic acid residues are missing from the carbohydrate side-chain.

The importance of having local pathology for the interpretation of biochemical data is emphasized by the results in this study. Although all the tissue specimens used for histological diagnosis showed signs of invasion, this observation was reported in only 5/11 of the adjacent specimens that were examined. Interestingly, the extent of loss of WGA-binding correlated with the local pathology. A similar type of discrepancy was found for patient 'd', who was placed in the 'benign' group from the evidence of local pathology; having been previously diagnosed as malignant. Biochemically, this extract gave a benign protein pattern, but a malignant glycoprotein pattern. This result suggests that

WGA-binding may be a more sensitive and reliable indicator of the presence of tumour than protein staining.

These results indicate intra-tumoral variability in the expression of cellular proteins, however, no significant variability was discovered within the samples of tissue that were available for biochemistry in this study (all data not shown, see 'u' Figure 2 for example). Intra-tumoral variability may have been detected if all the diseased tissue had been made available. Unfortunately, this was not the case, and the requirements for diagnostic histopathology obviously took precedence.

Several protein markers for human breast cancer have been reported and many of these can be monitored in tissue and body fluids by using monoclonal antibodies. The changes in glycosylation that we have detected with WGA suggest an alternative or additional approach to using these immunological reagents. Monoclonal antibodies only recognise molecules containing the epitope to which the antibody was raised, whereas WGA will recognise a whole host of different molecular species, provided that they contain certain sugars. WGA, therefore, may have wider application than monoclonal antibodies. Our results also suggest that the degree of reduced glycosylation could be correlated with the extent of tumour aggressiveness. Whether this change has a role to play in the spreading process itself, or is a by-product of it, is not known. Identifying the causes of spread are very important for developing rational programs of therapy. Further studies are necessary to establish the nature and source of the abnormal glycoproteins that we have detected in human breast cancer.

We gratefully acknowledge the staff at the Newcastle General Hospital, Newcastle upon Tyne for assistance in obtaining the tissue specimens and the GO fund, Durham and the North of England Cancer Research Campaign for financial support.

### References

BENSADOUN, A. & WEINSTEIN, D. (1976). Assay of proteins in the presence of interfering materials. *Anal. Biochem.*, **70**, 241.

BLOOM, H.J.G. & RICHARDSON, W. (1957). Histological grading and prognosis in breast cancer: a study of 1409 cases of which 359 have been followed for 15 years. *Br. J. Cancer*, **11**, 359.

BRAMWELL, M.E. & HARRIS, H. (1978). An abnormal membrane glycoprotein associated with malignancy in a wide range of different tumours. *Proc. R. Soc. Lond. B.*, **201**, 87.

BUTTERS, T.D. & HUGHES, R.C. (1974). Solubilization and fractionation of glycoproteins and glycolipids of KB cell membranes. *Biochem. J.*, **140**, 469.

CAPONY, F., GARCIA, M., VEITH, F. & ROCHEFORT, H. (1982). Antibodies to the estrogen induced 52K protein released by human breast cancer cells. *Biochem. Biophys. Res. Comm.*, **108**, 8.

CHAN, W-S., JACKSON, A. & TURNER, G.A. (1982). The loss of certain WGA-binding proteins in relationship to metastasis and to the site of tumour growth. *Br. J. Cancer*, **46**, 474.

CHAN, W-S., JACKSON, A. & TURNER, G.A. (1984). Differences in surface expression of WGA-binding proteins of cells from a lymphosarcoma and its liver metastases. *Br. J. Cancer*, **49**, 181.

CHAN, W-S., JACKSON, A. & TURNER, G.A. (1985). The binding of Con A and other lectins to surface glycoproteins. A comparison of a subcutaneous tumour and its liver metastases. *Invasion & Metastasis*, **5**, 233.

GARCIA, M., CAPONY, F., DEROCQ, D., SIMON, D., PAU, B. & ROCHEFORT, H. (1985). Characterisation of monoclonal antibodies to the oestrogen regulated Mr 52,000 glycoprotein and their use in MCF7 cells. *Cancer Res.*, **45**, 709.

GENDLER, S.J., DERMER, G.B., SILVERMAN, L.M. & TOKES, Z.A. (1982). Synthesis of $\alpha_1$-antichymotrypsin and $\alpha_1$-acidglycoprotein by human breast epithelial cells. *Cancer Res.*, **42**, 4567.

IRWIN, D. (1981). Protein patterns as codes for tumour identification. *Anal. Biochem.*, **118**, 392.

KHAN, H.J. & BAUMAL, R. (1985). Differences in lectin binding in tissue sections of human malignant tumors and their metastases. *Am. J. Pathol.*, **119**, 420.

SMETS, L.A. & VAN-BEEK, W.P. (1984). Carbohydrates of the tumor cell surface. *Biochem. Biophys. Acta.*, **738**, 237.

SPRINGER, G.F., DESAI, P.R. & SCANLON, E.F. (1976). Blood group MN precursors as human breast carcinoma associated antigens and naturally occurring human cytotoxins against them. *Cancer*, **37**, 169.

TURNER, G.A. (1982). Surface properties of the metastatic cell. *Invasion & Metastasis*, **2**, 197.

TURNER, G.A., CHAN, W-S. & JACKSON, A. (1984). Metastases and surface glycoproteins in relationship to tumour implantation site. *Br. J. Cancer*, **50**, 273.

TURNER, G.A. & CHAN, W-S. (1985). Reduced levels of WGA-binding proteins in metastatic nodules: a site modulated phenomena. In *Treatment of Metastasis: Problems and Prospects*, (ed) Hellmann, K. & Eccles, S.A. p 365. Taylor & Francis, London.

TURNER, G.A., SKILLEN, A.W., BUAMAH, P. & 4 others. (1985). Relationship between raised concentrations of fucose, sialic acid, and acute phase proteins in serum from patients with cancer: choosing suitable serum glycoprotein markers. *J. Clin. Pathol.*, **38**, 588.

WALKER, R.A. (1984). The binding of peroxidase-labelled lectins to human breast epithelium. II-The reactivity of breast carcinomas to wheat germ agglutinin. *J. Pathol.*, **144**, 101.

WILKINSON, M.J.S., HOWELL, A., HARRIS, M., TAYLOR-PAPADIMITRIOU, J., SWINDELL, R. & SELLWOOD, R.A. (1984). The prognostic significance of two epithelial membrane antigens expressed by human mammary carcinomas. *Int. J. Cancer*, **33**, 299.